# Enhancing Postharvest Quality and Shelf Life of Strawberries through Advanced Coating Technologies: A Comprehensive Investigation of Chitosan and Glycine Betaine Nanoparticle Treatments

**DOI:** 10.3390/plants13081136

**Published:** 2024-04-18

**Authors:** Reza Bahmani, Farhang Razavi, Seyed Najmmaddin Mortazavi, Gholamreza Gohari, Antonio Juárez-Maldonado

**Affiliations:** 1 Department of Horticulture, Faculty of Agriculture, University of Zanjan, Zanjan 45371-38791, Iran; bahmani720@gmail.com (R.B.); razavi.farhang@znu.ac.ir (F.R.); mortazavi46@znu.ac.ir (S.N.M.); 2 Department of Horticulture, Faculty of Agriculture, University of Maragheh, Maragheh 83111-55181, Iran; gohari.gh@maragheh.ac.ir; 3 Departamento de Botánica, Universidad Autónoma Agraria Antonio Narro, Saltillo 25315, Mexico

**Keywords:** antioxidant capacity, cold storage, nanocomposite, strawberry fruits

## Abstract

The application of natural polymer-based coatings presents a viable approach to prolong the longevity of fruits and tissue damage. This study investigates the impact of treatments involving glycine betaine (GB), chitosan (CTS), and chitosan-coated glycine betaine nanoparticles (CTS-GB NPs) on preserving the quality and reducing decay in strawberry fruits. The fruits were subjected to treatments with GB (1 mM), CTS (0.1%), CTS-GB NPs (0.1%), or distilled water at 20 °C for 5 min, followed by storage at 4 °C for 12 days. The results indicate that CTS and CTS-GB NPs treatments resulted in the highest tissue firmness, total anthocyanin content, and ascorbate peroxidase activity, while exhibiting the lowest decay percentage and weight loss, as well as reduced malondialdehyde levels at the end of storage. GB, CTS, and CTS-GB NPs treatments demonstrated elevated catalase activity and antioxidant capacity, coupled with lower electrolyte leakage and hydrogen peroxide levels. These treatments did not significantly differ from each other but were markedly different from the control. The results substantiate that CTS and CTS-GB NPs treatments effectively preserve strawberry quality and extend storage life by bolstering antioxidant capacity and mitigating free radical damage.

## 1. Introduction

The strawberry (*Fragaria* × *ananassa* Duchesne) belongs to the Rosaceae family and produces non-climacteric fruits whose quality is contingent upon factors such as appearance, texture, aroma, taste, and nutritional composition. Critical components influencing strawberry quality include sugars, amino acids, and aromatic compounds [1]. Despite being a significant horticultural crop, strawberries are highly perishable with a brief postharvest shelf life. Therefore, the imperative to discover practical approaches, particularly involving natural compounds, to extend their longevity is evident [2].

In recent decades, substantial efforts have been directed towards prolonging fruit longevity and preserving nutritional value during storage [3]. Utilizing coatings based on natural polymers is a pragmatic strategy to enhance fruit longevity and impede tissue damage. Edible coatings, particularly those derived from various polysaccharides such as cellulose, starch, and chitosan, have gained prominence in replacing synthetic counterparts in food packaging [4]. Chitosan, a natural polymer obtained through the deacetylation of crustacean, insect, or fungal chitin [5], has demonstrated potential in reducing weight loss and decay while augmenting antioxidant activity in fresh fruits [6,7,8].

Compatible organic compounds, such as proline, sorbitol, polyamines, and glycine betaine, are recognized for their role in enhancing osmotic pressure, thereby fortifying horticultural crops against abiotic stresses [9,10]. Glycine betaine, in particular, has exhibited protective effects against membrane damage due to temperature fluctuations in various crops, including pomegranates and cucumbers [9,11]. Despite its documented benefits, limited information exists regarding the postharvest application of glycine betaine to fruits and vegetables. Recent research has reported that the application of glycine betaine improved postharvest quality of some horticultural crops, e.g., cucumbers [12], grapes [13], papayas [14], peaches [15], pears [16], and pomegranates [17].

In recent times, nanotechnology has emerged as a transformative technology in agriculture, offering diverse applications across crop production, processing, storage, packaging, and transportation [18,19]. The unique properties of compounds at the nanoscale, with an increased surface-to-volume ratio, have proven advantageous in enhancing their effectiveness [20]. Chitosan, when incorporated into nanoparticles, has demonstrated efficacy in preserving the quality and longevity of fruits and vegetables [21,22,23,24]. Notably, chitosan nanoparticles, in combination with other compounds, have shown significant promise in preserving physicochemical quality and extending the storage life of bell peppers [25], apples [26], bananas [27], and green tomatoes [28]. Yu et al. [29] reported that the treatment of jujubes with chitosan combined with nano-silicon resulted in a decline in their quality traits, e.g., red color, respiration rate, decay percentage, and weight loss, over the 32 days of storage at room temperature versus the control. The application of chitosan-nanoparticle-coated glycine betaine to plums significantly mitigated chilling, preserved quality, and prolonged storage life [30]. However, no prior research has explored the impact of glycine betaine and chitosan nanocomposite-coated glycine betaine on postharvest quality in strawberries. Thus, this study aims to investigate the effects of glycine betaine, chitosan, and glycine betaine coated with chitosan nanocomposite (CTS-GB NPs) on decay control, enhancement of antioxidant enzyme activity, and improvement of strawberry quality during storage at 4 °C for 12 days.

## 2. Results and Discussion

### 2.1. Decay Percentage

**Figure 1:**
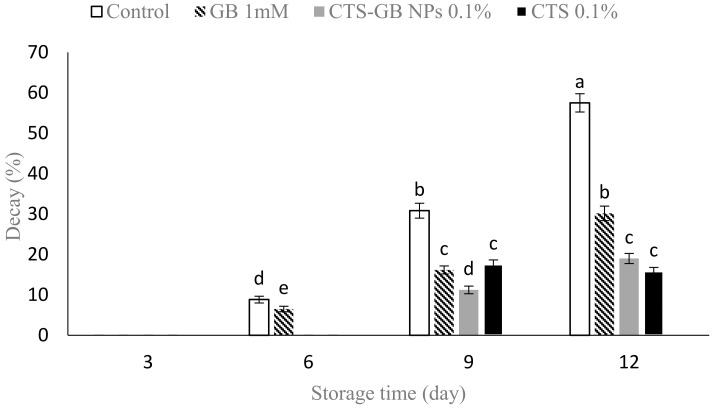
Impact of glycine betaine (GB), chitosan (CTS), and CTS-GB nanoparticles (CTS-GB NPs) on the decay percentage of strawberries over 12 days of storage. Data presented are mean ± standard error (SE) of three replications. Different letters over bars indicate they are significantly different (*p* < 0.05) by Duncan’s test.

Upon comparing means, no visible decay was observed in both the control and treated fruits after 3 days of storage. However, decay symptoms appeared in the control and GB-treated fruits on day 6. At the end of storage, the lowest decay percentage was observed in fruits treated with CTS-GB NPs or CTS, while the highest was noted in the control (Figure 1). GB and CTS treatments significantly prevented strawberry decay at the end of storage. Strawberries are highly perishable due to their high metabolic activity and susceptibility to fungal decay, particularly grey mold [31]. GB has been shown to reduce decay and enhance resistance to mold in apples by increasing CAT and SOD activity [32]. Similar positive effects on decay reduction were reported in sweet peppers [33] and apples [34], supporting our findings. The efficacy of CTS and nanocomposites in controlling decay has been highlighted in various fruits, including strawberries [35], grapes [36], and figs [37]. Notably, the CTS/cellulose nanofibril nanocomposite alleviated strawberry decay [38]. Similarly, the CTS/nano-TiO_2_ composite effectively reduced mango decay [39]. The antibacterial and antifungal activities of CTS, attributed to changes in cell permeability through interactions with cell surface charges, contribute to its effectiveness [40].

### 2.2. Weight Loss

**Figure 2:**
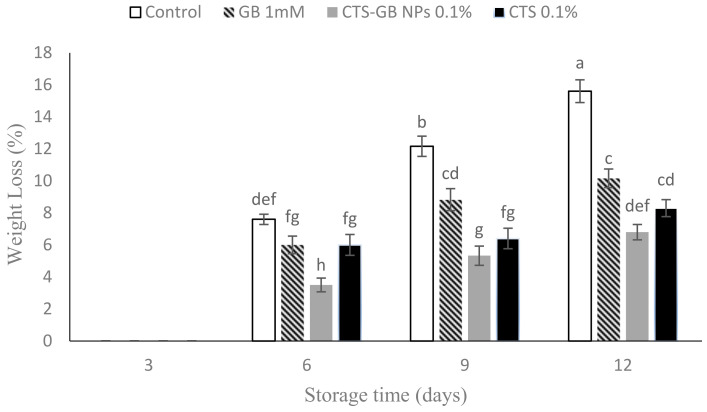
Effect of glycine betaine (GB), chitosan (CTS), and CTS-GB nanoparticles (CTS-GB NPs) on the weight loss of strawberries over 12 days of storage. Data presented are mean ± standard error (SE) of three replications. Different letters over bars indicate they are significantly different (*p* < 0.05) by Duncan’s test.

The results indicated an increase in weight loss for both treated and control fruits during storage, starting from day 6. However, CTS-GB-NP-treated fruits exhibited the lowest weight loss at the end of the storage period, followed by CTS-treated fruits, with the control showing the highest weight loss (Figure 2). GB, known for preventing dehydration and plasmolysis in high osmotic conditions [41], likely contributed to inhibiting weight loss during storage by regulating osmotic pressure and maintaining fruit moisture balance [42].

Research supports the effectiveness of nanocomposite and CTS coatings in inhibiting weight loss in various fruits, including mangoes [43], tomatoes [44], and kiwifruits [45]. A study on plums showed that CTS-GB NP coating reduced weight loss during 40 days of cold storage [30]. The coatings act as semi-permeable barriers, reducing transpiration and controlling O_2_ and CO_2_ exchange, thereby slowing down respiration [39].

### 2.3. Fruit Tissue Firmness

**Figure 3:**
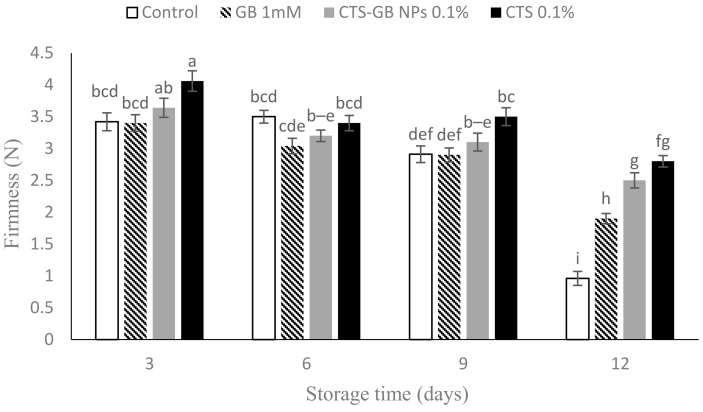
Influence of glycine betaine (GB), chitosan (CTS), and CTS-GB nanoparticles (CTS-GB NPs) on the tissue firmness of strawberries over 12 days of storage. Data presented are mean ± standard error (SE) of three replications. Different letters over bars indicate they are significantly different (*p* < 0.05) by Duncan’s test.

The firmness of fruit tissues decreased over the storage period (Figure 3). CTS-treated fruits showed the highest firmness on day 3, differing significantly from other treatments. At the end of storage, the highest firmness was observed in CTS- and CTS-GB-NP-treated fruits, significantly different with control and GB treatments. Loss of firmness is associated with cell wall changes, including hemicellulose and galactose loss and pectin dissolution, due to increased cell wall hydrolyzing enzyme activity [46]. GB positively affects enzyme synthesis and plasma membrane strength [47]. The preservation of firmness by CTS and CTS-GB NPs aligns with findings in plums [30], peppers [48], litchi fruits [49], apricots [50], tomatoes [51], and mangoes [52]. CTS likely reduces the activities of β-galactosidase, polygalacturonase, and pectin methylesterase, which are the most important enzymes that are responsible for cell wall degradation and fruit softening [26].

### 2.4. Total Soluble Solids (TSSs) and Titratable Acidity (TA)

**Figure 4:**
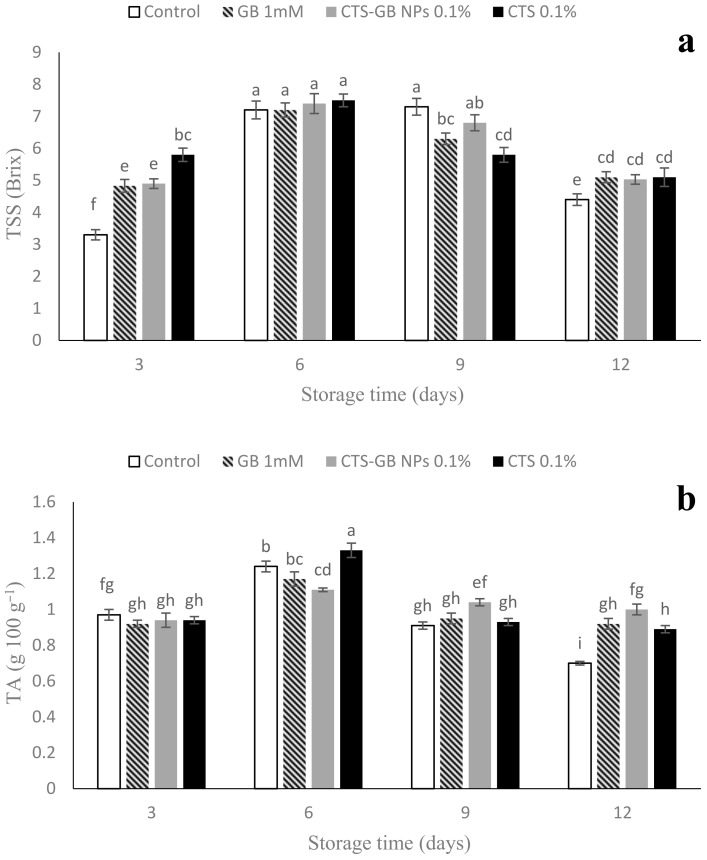
Impact of glycine betaine (GB), chitosan (CTS), and CTS-GB nanoparticles (CTS-GB NPs) on the total soluble solids (**a**) and titratable acidity (**b**) of strawberries over 12 days of storage. Data presented are mean ± standard error (SE) of three replications. Different letters over bars indicate they are significantly different (*p* < 0.05) by Duncan’s test.

TSSs exhibited an ascending trend until day 6, followed by a decline until the end of storage. The lowest TSSs at the end of the 12-day period were in the control, significantly different from other treatments. However, CTS, GB, and CTS-GB NPs did not significantly differ in TSSs (Figure 4a). TA was similar among treatments on day 3, increased until day 6, and then declined. At the end, CTS-GB NPs and CTS exhibited the highest TA (Figure 4b). Loss of organic acids during storage may relate to metabolic changes or acid consumption for respiration [50]. GB has been reported to increase TSSs and TA in sweet peppers [53] and cherries [54]. CTS contributes to preserving TSSs and TA in litchi fruits [55]. The effects of exogenous GB on TSSs and TA can be attributed to changes in internal fruit atmosphere, reducing respiration and converting starch into sugar [54]. CTS and CTS nanocomposite coatings increased TSSs and TA in mushrooms and peppers [56,57], supporting our findings.

### 2.5. Ascorbic Acid

**Figure 5:**
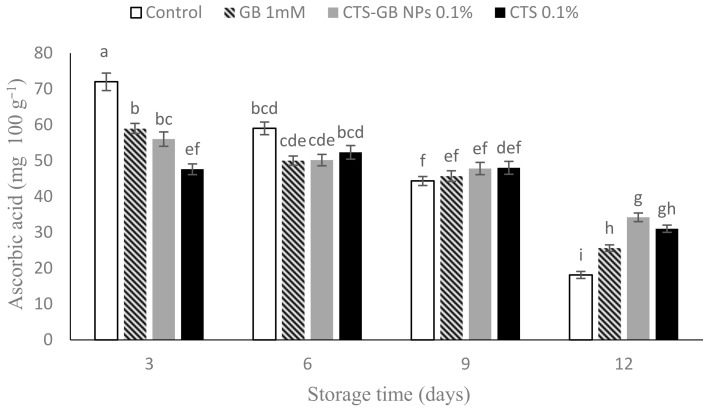
Effect of glycine betaine (GB), chitosan (CTS), and CTS-GB nanoparticles (CTS-GB NPs) on the ascorbic acid content of strawberries over 12 days of storage. Data presented are mean ± standard error (SE) of three replications. Different letters over bars indicate they are significantly different (*p* < 0.05) by Duncan’s test.

Ascorbic acid content decreased during storage, with the lowest observed in the control at the end of storage. Treatments inhibited excessive ascorbic acid loss, with CTS-GB NPs being the most effective, followed by CTS and GB (Figure 5). GB contributes to ascorbic acid preservation by inhibiting cell wall degradation and reducing reactive oxygen species (ROS) [58]. GB enhances the activity of enzymes involved in ascorbic acid production [59]. Similar preservation effects have been reported in bananas, sweet peppers, and cherries [33,60,61].

### 2.6. Electrolyte Leakage (EL), Malondialdehyde (MDA), and Hydrogen Peroxide (H_2_O_2_)

**Figure 6:**
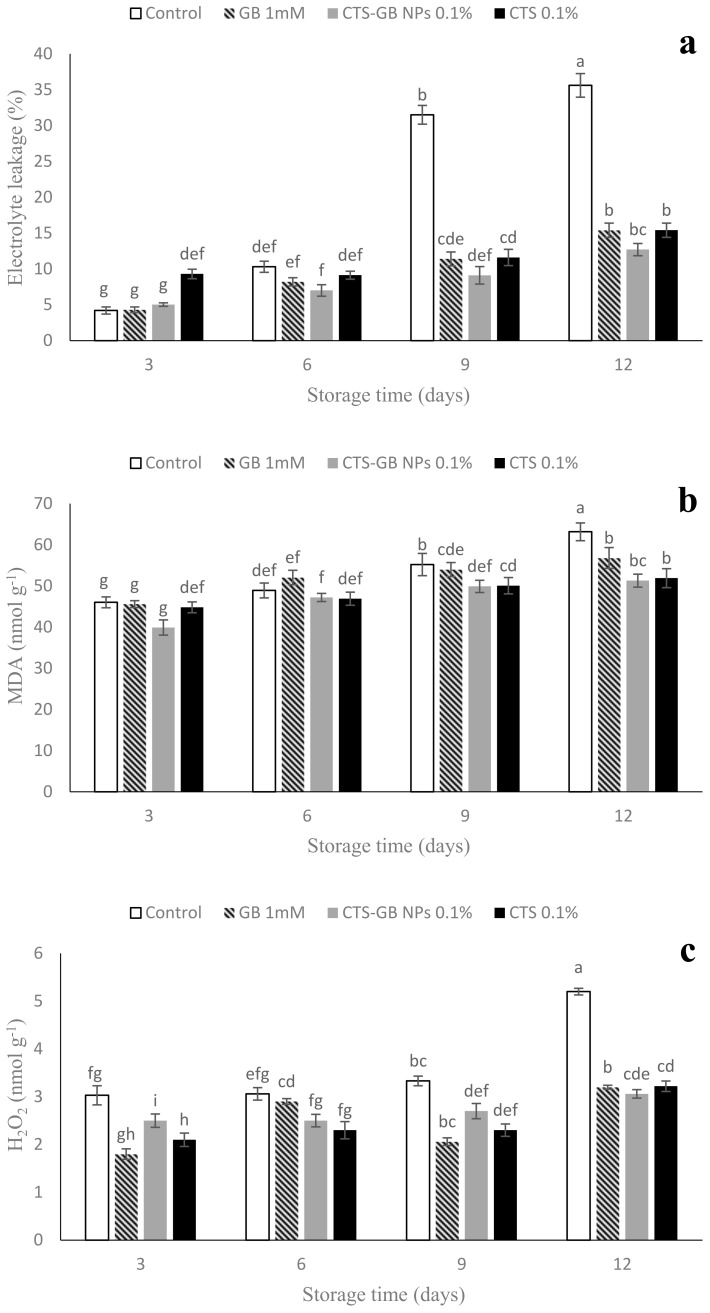
Impact of glycine betaine (GB), chitosan (CTS), and CTS-GB nanoparticles (CTS-GB NPs) on the electrolyte leakage (**a**), malondialdehyde (**b**) content, and hydrogen peroxide (**c**) of strawberries over 12 days of storage. Data presented are mean ± standard error (SE) of three replications. Different letters over bars indicate they are significantly different (*p* < 0.05) by Duncan’s test.

EL increased in all strawberries during storage, particularly in the control. CTS, CTS-GB NPs, and GB effectively prevented EL increase at the end of storage (Figure 6a). Fruits accumulated MDA during storage, with CTS and CTS-GB NPs showing significantly lower MDA content compared to the control and GB (Figure 6b). H_2_O_2_ remained stable until day 9, increasing thereafter. At the end of storage, the highest H_2_O_2_ was in the control and the lowest in GB-, CTS-, and CTS-GB-NP-treated fruits, significantly different from the control (Figure 6c). GB protects membrane integrity in soybeans by reducing EL [9]. Membrane fat peroxidation is the first symptom of chilling damage in plants, which is revealed with the increase in MDA accumulation. Researchers consider the fat peroxidation level a plant mechanism against various stresses [62].

It has been proven that the main reason for the severe damage to cell membranes is the synthesis of hydroxyl, superoxide, and H_2_O_2_ radicals, which leads to the peroxidation of unsaturated fats in cell membranes [63]. Higher GB concentrations contribute to membrane stability and structure by reducing the peroxidation of unsaturated fats in cell membranes during stress [63]. Similar reductions in EL and MDA have been reported in loquats, pomegranates, and plums [17,30,64]. CTS and CTS-GB NPs also effectively scavenge free radicals, reducing EL and MDA in bananas, longans, and kiwifruits [18,27,45]. The levels of EL and MDA, along with H_2_O_2_ content, showed an increase throughout the cold-storage duration, indicating the onset of oxidative stress due to elevated ROS production stemming from cell membrane damage [30]. This observation is consistent with the pronounced EL levels observed in our study. The reduction in H_2_O_2_ levels in strawberry fruits during cold storage can be attributed to the heightened activity of both enzymatic and non-enzymatic antioxidants, which are critical in scavenging H_2_O_2_. This phenomenon is confirmed by the findings of Mahmoudi et al. [30], who reported that GB and CTS-GB NPs treatments contributed to the alleviation of H_2_O_2_ accumulation in plus fruits during the cold storage.

### 2.7. Total Anthocyanin Content

**Figure 7:**
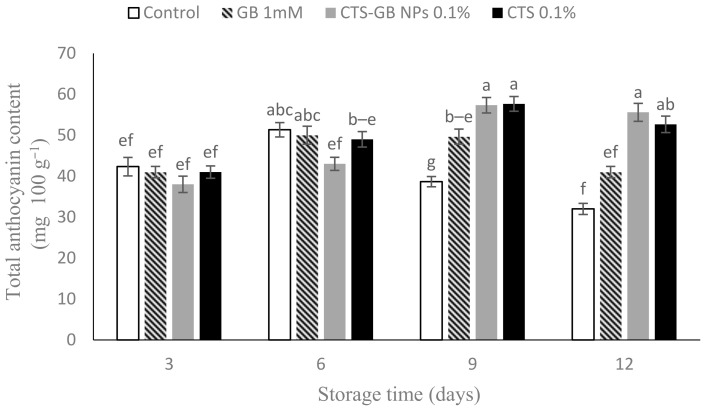
Influence of glycine betaine (GB), chitosan (CTS), and CTS-GB nanoparticles (CTS-GB NPs) on the anthocyanin content of strawberries over 12 days of storage. Data presented are mean ± standard error (SE) of three replications. Different letters over bars indicate they are significantly different (*p* < 0.05) by Duncan’s test.

Anthocyanin content increased over the storage period, peaking on day 9, and then declined until the end. CTS-GB-NP- and CTS-treated fruits showed the highest anthocyanin content, significantly different from other treatments (Figure 7).

Strawberry fruits are rich in anthocyanin. The dominant anthocyanin is pelargonidin 3-glucoside [65]. The anthocyanin content increases during the postharvest period due to its biosynthesis, but it begins to decline during storage due to the aging process [66]. Anthocyanin preservation by CTS has been reported in pomegranates, litchis, and raspberries [67,68,69]. CTS-GB NPs also preserved anthocyanin content in plums [30]. Coatings likely slow maturity, reduce oxygen levels, and inhibit peroxidase and polyphenol oxidase enzyme activity, preserving anthocyanins [7].

### 2.8. Total Phenol, Flavonoid, and Antioxidant Capacity

**Figure 8:**
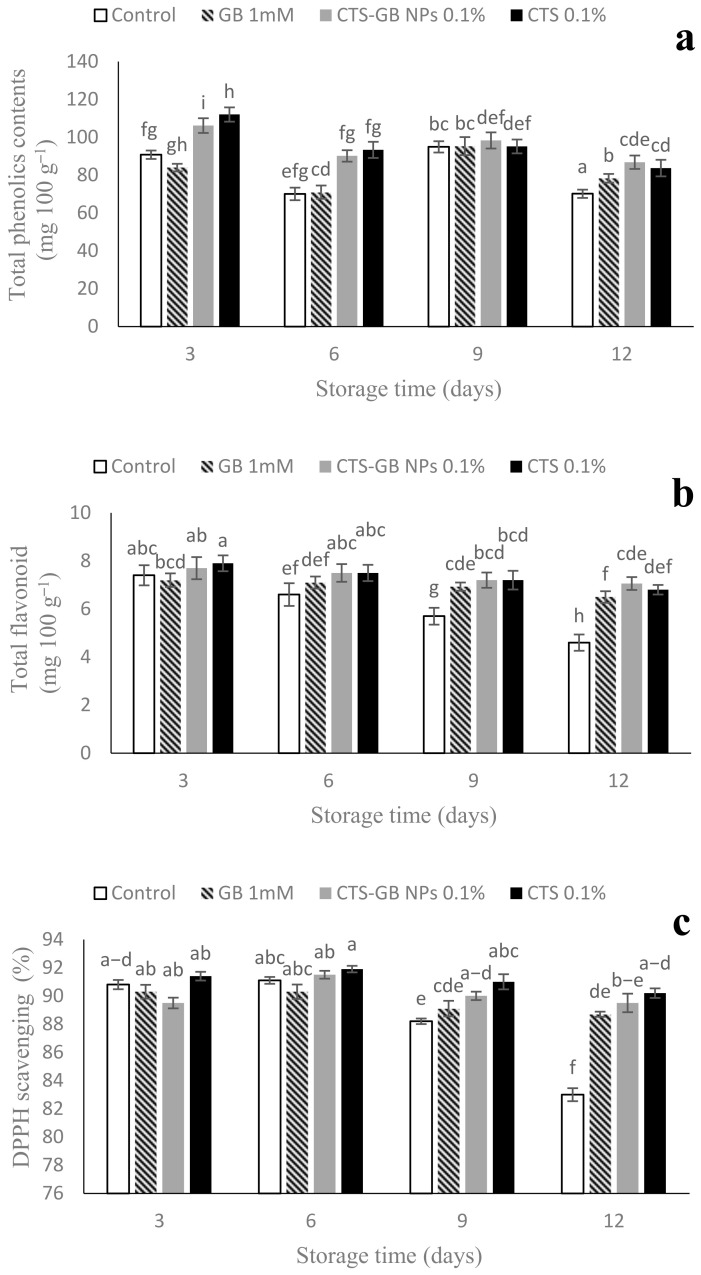
Effect of glycine betaine (GB), chitosan (CTS), and CTS-GB nanoparticles (CTS-GB NPs) on the phenols (**a**), flavonoids (**b**), and total antioxidants (**c**) of strawberries over 12 days of storage. Data presented are mean ± standard error (SE) of three replications. Different letters over bars indicate they are significantly different (*p* < 0.05) by Duncan’s test.

Total phenol content decreased during storage, with the highest content in CTS-GB-NP- and CTS-treated fruits on days 3 and 6. At the end of storage, the highest was observed in the fruits treated with CTS-GB NPs, differing from the CTS treatment insignificantly but from the control and GB treatment significantly (Figure 8a).

Total flavonoid content decreased in all treated fruits during storage. The treatments did not develop significant differences in this trait from day 3 to day 9, but they differed from the control significantly. At the end of storage, the highest total flavonoid was obtained from the treatment of CTS-GB NPs, differing from the control and GB treatment significantly but from the CTS treatment insignificantly (Figure 8b). Antioxidant capacity exhibited a descending trend from day 6, with CTS-GB-NP-treated fruits showing the highest capacity at the end, significantly different from the control but not from other coating treatments (Figure 8c).

Phenols play a crucial role in scavenging free radicals and preventing H_2_O_2_ conversion into free radicals. As fruits ripen, extensive changes happen in gene expression, the activity of enzymes, and the synthesis of phenolic metabolites [70]. CTS, its nanocomposite, and CTS-GB NPs have been shown to increase total phenols, flavonoids, and antioxidants in mangoes and plums [30,39]. Strawberry coatings with nanocomposites have preserved more phenols and flavonoids than the control during storage [38]. The decline in total phenol content is attributed to oxidative damage and polyphenol oxidase activity, which CTS can counteract by stimulating the phenylpropanoid pathway [39]. In the present work, the total phenol and flavonoid contents were preserved in all treatments better than in control over storage, showing the effectiveness of coating treatments in preserving phenols and flavonoids.

### 2.9. Antioxidant Enzymes Activities

**Figure 9:**
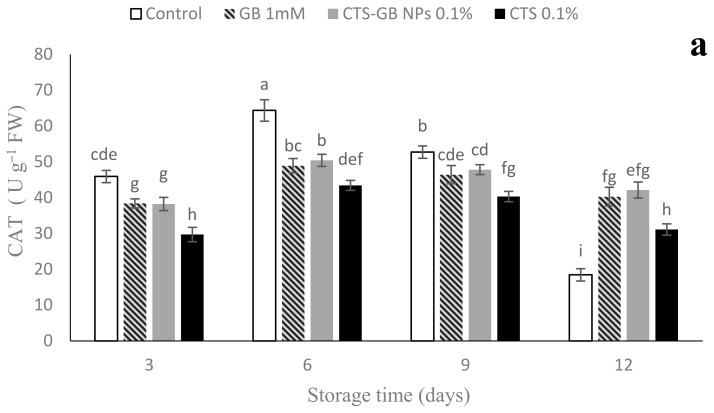

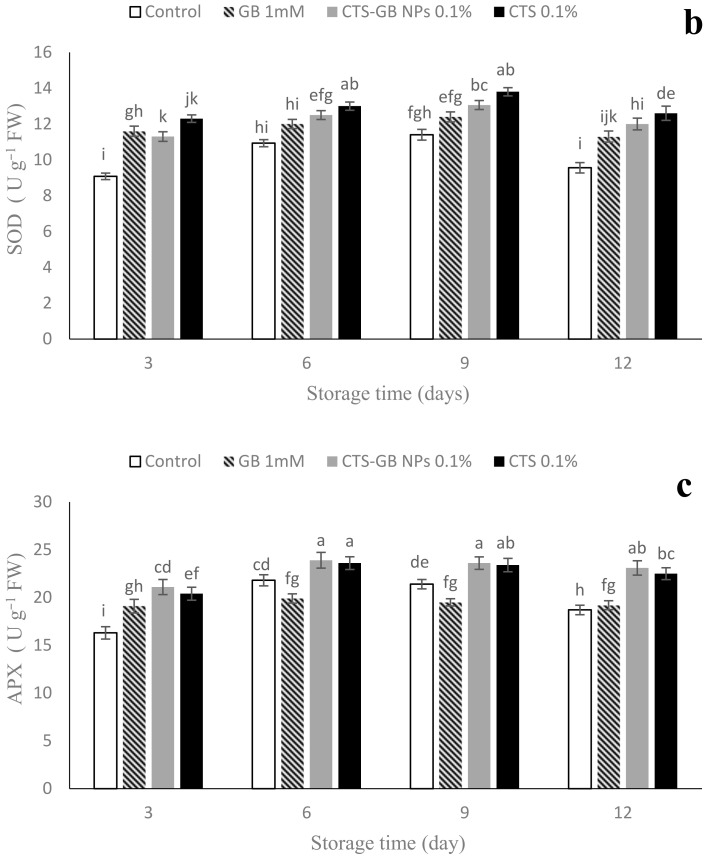
Influence of glycine betaine (GB), chitosan (CTS), and CTS-GB nanoparticles (CTS-GB NPs) on the catalase (**a**), superoxide dismutase (**b**), and ascorbate peroxidase (**c**) of strawberries over 12 days of storage. Data presented are mean ± standard error (SE) of three replications. Different letters over bars indicate they are significantly different (*p* < 0.05) by Duncan’s test.

CAT activity increased and then decreased in all samples during storage. The highest CAT activity was related to the control on days 6 and 9; CTS-GB-NP- and GB-treated fruits effectively increased CAT activity at the end, significantly different from the control and CTS (Figure 9a). SOD activity gradually increased until day 9, but then decreased until the end of storage. On day 9, the treatments of CTS-GB NPs and CTS effectively inhibited the loss of SOD activity. In the end, the SOD activity was the highest in the treatment of CTS, which differed from the other coating treatments and the control significantly. The lowest was related to the control (Figure 9b). APX activity was the highest in CTS-GB-NP- and CTS-treated fruits, effectively preventing its decline at the end of storage (Figure 9c). Antioxidant enzymes, including SOD, CAT, and APX, play a crucial role in suppressing free radicals and enhancing chilling tolerance in some chilling-sensitive fruits, such as mangoes [36], peaches [71], and loquats [64]. Higher activity of antioxidant enzymes and lower H_2_O_2_ content in GB- and CTS-GB-NP-treated strawberries suggest their role in preserving membrane health and structure by reducing ROS effects [32]. Similar effects of CTS and CTS-GB NPs have been observed in loquats, pomegranates, and plums [17,30,64]. Coatings with CTS, nanocomposites, and CTS-GB NPs have effectively preserved antioxidant activity in grapes, tomatoes, and cucumbers [28,36,72].

## 3. Materials and Methods

### 3.1. Plant Material, Trial Location, and Time

The research was conducted on strawberry cv. ‘Camarosa’ fruits in a greenhouse in Zanjan, Iran in 2020. The fruits were harvested at the commercial maturity stage (>80% of the surface red color) and were selected in terms of uniform in shape and size and absence of visual damage. Then, they were immediately transferred to the postharvest physiology laboratory of the Department of Horticulture, University of Zanjan to be applied with the treatments.

### 3.2. Nanocomposite Preparation

The nanomaterials were synthesized at the Laboratory of Nanochemistry, University of Maragheh, Maragheh, Iran, according to Bahmani et al. [23] and Mahmoudi et al. [30].

### 3.3. Application of Treatments

Strawberries underwent treatments by immersing in solutions containing 1 mM glycine betaine, 0.1% (*w*/*v*) CTS, 0.1% (*w*/*v*) CTS-GB NPs, or deionized water (control) at 20 °C for 5 min. Subsequently, the treated fruits were placed on kraft paper and allowed to dry for 1 h at room temperature (20 °C). Following drying, the fruits were sealed in polyethylene film bags of 0.03 mm in thickness with 4 to 5 holes (7–8 mm) to maintain the composition of air within the container and stored in a cold-storage facility at 4 °C with a relative humidity of 85–90% for 12 days. They were randomly allocated into five groups of 240 for each treatment in 3 replications (80 per replication). There were three boxes from each treatment, serving as three replications. The samples were taken out of storage on days 3, 6, 9, and 12, and their shelf life was evaluated after 24 h at 20 °C. After that, samples of fruit from each treatment/replicate were collected for quality measurements. Also, the additional samples of fruits were frozen in liquid nitrogen and stored at −80 °C immediately. The frozen samples were used to determine the content of ascorbic acid, malondialdehyde (MDA), hydrogen peroxide (H_2_O_2_), flavonoids, total anthocyanin, total phenolic, total antioxidant capacity, and the antioxidant activity of catalase (CAT), superoxide dismutase (SOD), and ascorbate peroxidase (APX).

### 3.4. Decay Percentage

The decayed fruits by storage rot were recorded. The decay percentage was calculated from the initial fruit number for each sample and expressed as a percentage.

### 3.5. Weight Loss Percentage

Weight loss was determined by weighing the strawberries before the experiment and after storage [73]. The percentage of weight loss was calculated using the following formula:

(Weight before storage − Weight on sampling day)/Weight before storage × 100.

### 3.6. Fruit Tissue Firmness

Tissue firmness was measured using a handheld hardness tester (FT011, Facchini srl, Alfonsine (Ra), Italy) with a 5 mm probe, and the results were expressed in newtons (N).

### 3.7. Total Soluble Solids (TSSs) and Titratable Acidity (TA)

The TSSs were measured with a handheld refractometer (PAL-1, Atago Co., Tokyo, Japan). Titratable acidity (TA) was determined by titrating 10 mL of fruit juice with sodium hydroxide 0.1 N until the extract’s pH reached 8.1 [74].

### 3.8. Ascorbic Acid Content

Ascorbic acid content was determined through titration with potassium iodide, and the results were expressed as mg 100 g^−1^ fresh weight [74].

### 3.9. Anthocyanin Content

Anthocyanin content was determined using the pH differential method. A mass of 1 g of fruit tissue was added to 10 mL of methanol containing hydrogen chloride 1% and was kept at 0 °C for 10 min. Then, 200 µL of the supernatant was added to 1800 µL of potassium chloride buffer (pH = 1) and 1800 µL of acetate sodium buffer (pH = 4.5). Finally, absorbance was recorded at 510 and 700 nm, and the TAC was calculated in mg per 100 g of fresh weight [73].

### 3.10. Total Phenol and Flavonoid Content, and Antioxidant Capacity

Extraction of phenols, flavonoids, and total antioxidant capacity was performed by grinding 1 g of fruit tissue with methanol 80%.

Total phenol content was determined using the Folin–Ciocalteu reagent. For this, 100 µL of the extract was combined with 2 mL of 2% sodium carbonate in a test tube. After 5 min, 100 µL of diluted Folin–Ciocalteu reagent (50%) was added. After that, the mixture was kept in the dark at room temperature for 30 min, and the absorbance was measured at 720 nm with the Specorp 250 Jena-History spectrophotometer (Analytik Jena GmbH+Co. KG, Jena, Germany). The standard curve was generated using different concentrations of gallic acid, and total phenol content was reported in mg 100 g^−1^ FW [74].

For total flavonoid content determination, 250 µL of the extract was mixed with 75 µL of 5% sodium nitrite, 150 µL of 10% aluminum chloride, and 500 mL of 1 M sodium hydroxide. It was then adjusted to 2.5 mL with distilled water. After 5 min, absorbance was read at 507 nm using a Spercorp 250 Jena-History spectrophotometer (Analytik Jena GmbH+Co. KG, Jena, Germany). The standard curve was constructed based on absorbance at specific quercetin concentrations.

Antioxidant capacity was determined using the DPPH method. A 0.1 mM DPPH solution was prepared, and 50 µL of the fruit extract was added to 1950 µL of the DPPH solution (0.1 mM) to reach a final volume of 2 mL. Absorbance was read at 517 nm with the Specorp 250 Jena-History spectrophotometer (Analytik Jena GmbH+Co. KG, Jena, Germany) after 20 min. The DPPH solution was used as a reference to compare absorbance, and antioxidant capacity was calculated as a percentage using the following equation [74]:

DPPH scavenging capacity (%): (Ac − As/Ac) × 100

where Ac is the absorbance of the control, and As is the absorbance of the sample.

### 3.11. Malondialdehyde (MDA) and Hydrogen Peroxide (H_2_O_2_) Content

For MDA estimation, 1 g of fruit tissue was mixed with 5 mL of 10% trichloroacetic acid solution and centrifuged at 10,000 rpm for 5 min. Then, 2 mL of the supernatant was mixed with 2 mL of 1% trichloroacetic acid and 0.6 g of thiobarbituric acid. The mixture was heated in a water bath at 100 °C for 20 min, quickly cooled in ice, and re-centrifuged at 10,000 rpm for 5 min. Absorbance was recorded at 532 and 600 nm, and the results were presented in nmol g^−1^.

MDA = (A_532_ − A_600_) × W × V/155 × 1000

where W is the weight of fruit tissue, and V is the extract volume.

To measure hydrogen peroxide, 1 g of fruit tissues was ground in a china mortar containing 5 mL of 1% trichloroacetic acid. The resulting extract was centrifuged at 10,000 rpm at 4 °C for 5 min. Then, 750 µL of the supernatant was mixed with 250 µL of 10 mM potassium phosphate buffer (pH = 7) and 1 mL of 1 M potassium iodide. The absorbance was read at 390 nm with a spectrophotometer. H_2_O_2_ content was determined in nmol g^−1^ using the standard curve [75].

### 3.12. Antioxidant Enzymes Activity

To assess the activity of antioxidant enzymes, including superoxide dismutase (SOD) and catalase (CAT), 1 g of fruit tissue was ground with 5 mL of 50 mM potassium phosphate (KH_2_PO_4_) (pH = 7.8) containing 0.2 mM Na_2_EDTA and 2% (*w*/*v*) polyvinylpolypyrrolidone (PVP). The homogenous solution was centrifuged at 12,000 rpm at 4 °C for 20 min, and the supernatant was used as the enzymatic extract.

#### 3.12.1. Catalase (CAT) Activity

To determine CAT activity, 100 µL of the enzymatic extract was combined with 50 µL of hydrogen peroxide (H_2_O_2_) and 200 µL of 50 mM phosphate buffer (pH = 7). One unit of CAT activity was recorded as the decline in absorbance at 240 nm over two minutes. The results were expressed in U g^−1^ FW [76].

#### 3.12.2. Superoxide Dismutase (SOD) Activity

For SOD activity measurement, 50 µL of the enzymatic extract was mixed with 2 mL of 50 mM potassium buffer, 100 µL of ethylenediaminetetraacetic acid (EDTA), and 200 µL of nitroblue tetrazolium test (NBT). The mixture was exposed to 40 W fluorescent light for 10 min. Then, 50 µL of 0.15 mM riboflavin was added, and the mixture was exposed to the same light for 12 min. The absorbance was read at 560 nm. SOD activity was expressed in U g^−1^ FW based on the amount of enzyme that inhibited NBT oxidation by 50% [76].

#### 3.12.3. Ascorbate Peroxidase (APX) Activity

To measure APX activity, 100 µL of the enzymatic extract was mixed with 2.9 mL of 0.5 mM ascorbic acid reaction solution, 1 mM H_2_O_2_, and 50 mM phosphate buffer. One unit of APX activity was defined as the amount of the enzyme that oxidized 1 µM ascorbate and was recorded in U g^−1^ FW based on the decline in absorption at 290 nm over one minute [76].

### 3.13. Experimental Design and Data Analysis

The study employed a factorial experiment with a completely randomized design, involving three replications. The first factor comprised the treatments (1 mM GB, 0.1% CTS, 0.1% CTS-GB NPs, and control), while the second factor was time. Data analysis was conducted using SPSS software (ver. 22), and means were compared using Duncan’s test at a significance level of *p* < 0.05.

## 4. Conclusions

In conclusion, our study demonstrates the effectiveness of chitosan and glycine betaine nanoparticle coatings in enhancing the postharvest quality of strawberries. These coatings significantly reduced decay, minimized weight loss, and preserved key attributes, including firmness and biochemical content. The nanocomposite formulation further enhanced these effects. Overall, our findings highlight the potential of these coatings as a sustainable and efficient postharvest treatment, offering improved quality and extended shelf life for strawberries.

## Data Availability

Data are contained within the article.
